# Large positive magnetoresistance and Dzyaloshinskii–Moriya interaction in CrSi driven by Cr 3*d* localization

**DOI:** 10.1038/s41598-020-67617-y

**Published:** 2020-07-21

**Authors:** Soma Banik, M. K. Chattopadhyay, Shilpa Tripathi, R. Rawat, S. N. Jha

**Affiliations:** 1 Synchrotron Utilization Section, Raja Ramanna Centre for Advanced Technology, Indore, 452013 India; 2 Homi Bhabha National Institute, Training School Complex, Anushakti Nagar, Mumbai, 400094 India; 3 Free Electron Laser Utilization Laboratory, Raja Ramanna Centre for Advanced Technology, Indore, 452013 India; 4 Atomic and Molecular Physics Division, Bhabha Atomic Research Center, Mumbai, 400085 India; 5 UGC-DAE Consortium for Scientific Research, Khandwa Road, Indore, 452001 India

**Keywords:** Materials science, Physics

## Abstract

Spin chiral systems with Dzyaloshinskii–Moriya (*DM*) interaction due to broken inversion symmetry are extensively studied for their technological applications in spintronics and thermoelectrics. Here, we report an experimental study on the magnetization, magnetoresistance (*MR*) and electronic structure of a non-centrosymmetric compound CrSi with B20 crystal structure. Both magnetization and *MR* shows competing ferromagnetic (*FM*) and antiferromagnetic (*AFM*) correlations with the *FM* correlations being comparatively weaker indicating the presence of *DM* interaction in CrSi. A large positive *MR* $\sim \,25\%$ obtained at 5 K and 5 T magnetic field arises due to the stronger *AFM* correlations. Resonant photoemission shows both localized and itinerant nature of Cr 3*d* electrons to be present in CrSi and this is supported by the temperature dependence of magnetic susceptibility. Drastic variation in the density of states along with valence band broadening at low temperature indicates the increase in hybridization between Cr 3*d* and Si 3*s*–3*p* states which enhances the localization effects. Spin polarized itinerant Cr 3*d* electrons give rise to *AFM* spin density wave in CrSi. Magnetic interaction between the localized and itinerant Cr 3*d* electrons are found to be crucial for realizing DM interaction in this system. Spectral density of states derived from high resolution valence band measurements provides evidence of electronic topological transition in CrSi. Large density of polarized itinerant electrons which varies with temperature and the large positive *MR* with *AFM* correlations suggests CrSi as a potential candidate for both the thermoelectric and spintronics applications.

## Introduction

Materials with chiral magnetism have attracted immense interest in the field of spintronics and thermoelectrics due to their non-trivial response to external magnetic field, ultrafast dynamics, large magnetotransport and spin Seebeck effect^1–6^. Spin chirality arises due to the competition between the Heisenberg exchange and the Dzyaloshinskii–Moriya (*DM*) interaction^7–9^. *DM* interaction occurs when inversion symmetry is broken at the interfaces or in the volume of non-centrosymmetric materials and generates twisted spin alignments^7,9^. *DM* interaction is understood in the localized moment picture with strong spin orbit coupling and broken inversion symmetry though its presence is known to give rise to weak itinerant magnetism^7–9^. Broken inversion symmetry is quite obvious in the noncentrosymmetric B20 transition metal monosilicites and monogermanides like MnSi, FeGe etc.^10,11^ where the presence of *DM* interaction is reported. However, it is found that broken inversion symmetry does not always give rise to *DM* interaction as it is not reported in other systems with B20 crystal structure. For example, FeSi is reported to be a non magnetic semiconductor^12^, CoSi is a non magnetic semimetal^13,14^ and NiSi is a non magnetic metal^15^. In this series, CrSi has been recently speculated to exhibit * DM * interaction and a chiral magnetic structure^16^. However, a detailed investigation on the magnetic and electronic properties of CrSi are not available in literature. CrSi also belongs to the category of metallic thermoelectric compounds where the change in the density of states at the Fermi level with temperature is reported to cause a change in the Seebeck coefficient^17^. CrSi thick films have important applications in magnetoresistive memories^18^. It has been reported that CrSi not only provides a good barrier to oxygen, its bulk resistivity is also low enough to provide sense line contact^18^.

Spin-dependent transport is an extremely important aspect in these chiral systems and magnetoresistance (*MR*) is a frequently used tool to study the same. A large positive *MR* has been reported in Cr metal^19^ and other Cr based system like $\hbox {Cr}_2\hbox {NiGa}$^20^, Cr/Ag/Cr trilayers^21^ etc. Cr metal is a prototype of the itinerant antiferromagnetism associated with the spin density wave (SDW)^22^. The SDW in Cr alloys is produced by the Coulomb attraction between electrons and holes on almost perfectly nested Fermi surface^22^. Doping in Cr with Si is reported to change the amplitude of the SDW and the relative sizes of the nesting on the Fermi surface^23,24^. However, the possibility of SDW in stoichiometric CrSi and the spin-dependent transport has not been explored experimentally as well as theoretically.

The potential for technological applications of CrSi may be realized only after proper understanding of its magnetic and electronic behaviour. In the present work, we have therefore performed detailed studies on the magnetization (*M*), magnetoresistance (*MR*) and electronic structure of CrSi. Both *M* and *MR* as a function of temperature (*T*) and field (*H*) indicated that CrSi is a compound with mixed magnetic interactions, the details of which are depicted in our results. We find that CrSi exhibits a large positive *MR* at low temperatures which increases to $\sim \,25\%$ at 5 K in 5 T magnetic field. The *M* and *MR* results showed itinerant electron magnetic behaviour with strong antiferromagnetic correlations indicating the presence of the SDW in CrSi which is further confirmed by the photoemission measurements. In general, SDW instabilities in a local moment system is underestimated in density functional theory because the Coulomb correlation considered in the calculation to generate the localized states causes suppression of the itinerant electronic states^25^. Hence, it is necessary to determine the density of both localized and itinerant states experimentally to understand the mechanism behind magnetism in CrSi. In this regard, resonant photoemission (RPES) is found to be an extremely powerful tool to experimentally determine the partial density of itinerant and localized states^1,26,27^. Hence, we have performed the RPES studies of Cr 3*d* states in CrSi to understand and explain the possible reasons for the origin of weak itinerant magnetism and *DM* interaction in this system.

**Figure 1:**
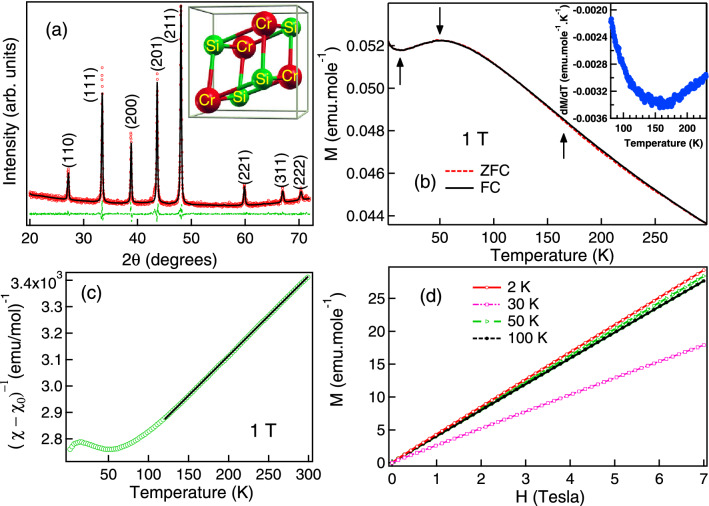
X-ray diffraction pattern of CrSi recorded using Cu $\hbox {K}_\alpha$ source is shown in (**a**). The experimental data is denoted by open red circle, while the black solid line represents the calculated pattern obtained by Le Bail decomposition and the green dashed line represents the difference between the experimental data and the fitting. The B20 crystal structure of CrSi is shown in the inset of (**a**). In (**b**), the Zero Field Cooled (ZFC) and Field Cooled (FC) magnetization (almost indistinguishable) measurement of CrSi under applied magnetic field of 1 T are shown. This temperature dependence of magnetization exhibits an inflection point near 165 K. The inflection point is determined with the help of the minimum in the first derivative of magnetization (*dM*/*dT*) shown in the Inset of (**b**). In (**c**) the temperature dependence of 1/$(\chi -\chi _0)$ for CrSi determined from the ZFC magnetization curve of 1 T is shown. The solid black line in (**c**) is the fitting using Eq. (1) which deviates from the experimental points below 125 K. In (**d**) the *M* versus *H* plots at different temperatures are shown.

## Results and discussions

### Structural information

The XRD pattern of CrSi is shown in Fig. 1a. Analysis of the XRD pattern indicates that CrSi has cubic B20 crystal structure with non-centrosymmetric P213 (198) space group as shown in the inset of Fig. 1a. The lattice parameter determined from Le Bail fitting, $a= 4.629\, \AA$, is in good agreement with the literature^28^. Presence of any impurity phase is not observed in the XRD pattern. However, there is preferred orientation in the data as the pattern has been collected on a bulk pellet.

### Magnetization and magnetoresistance behaviour

Figure 1b shows the *M* versus *T* curve for CrSi measured in 1 T magnetic field. CrSi shows a paramagnetic (*PM*) behaviour at high *T*, from 300 K down to about 165 K. Near 165 K it exhibits an inflection point marked in the figure with a up-arrow. The presence of the inflection point is confirmed by the corresponding minimum in the first derivative of magnetization (*dM*/*dT*) shown in the Inset to Fig. 1b. Such an inflection point generally indicates a *PM* to *FM* phase transition. However, unlike a *FM* material, the *M*(*T*) curve shows a broad maximum at about 50 K (marked by a down-arrow in Fig. 1b) and then exhibits a shallow minimum close to 15 K (second upward arrow in Fig. 1b). We have found that the $\chi (T)$ of CrSi cannot be analyzed solely by the Curie–Weiss law^8^. At high *T*, the $\chi (T)$ may be fitted with the following equation:

1 $$\chi = \chi _0 + \frac{C}{T-\theta }$$

Here $$\chi _0$$ is the temperature-independent part of the susceptibility, *C* is the Curie–Weiss constant and $$\theta$$ is the characteristic Curie–Weiss temperature. The constant *C* gives information about the magnitude of the Cr moments. Figure 1c shows the ($$\chi -\chi _0$$)$$^{-1}$$ versus *T* plot of CrSi, where a clear deviation from linearity is observed below 125 K. In the presence of the inflection point near 165 K, this deviation from linearity was expected below this latter temperature. We believe that this difference of temperatures is related to the very slow change of slope of the *M*(*T*) curve around the inflection point (evident from the rather shallow minimum in the *dM*/*dT* curve shown in the inset to Fig. 1b). The curve fitting at higher *T* (165–300 K) yields $$\chi _0 = 5.61 \times 10^{-5}$$ emu/mol, $$C = 0.332$$ emu K/mol, and $$\theta =-\,833$$ K as the best fit parameters. While the value of $$\chi _0$$ is small as compared to $$\chi$$, we have checked that this value is not due to the temperature independent background contributed by the experimental set-up (MPMS-3 SQUID VSM, resolution $$10^{-9}$$ emu). The signal range for the CrSi sample during this measurement (Fig. 1b) was $$3\times 10^{-4}$$ emu (+ve) and the background level was $$- (10^{-6}$$–$$10^{-7})$$ emu (diamagnetic). The finite value of $$\chi _0$$ indicates the itinerant electron magnetic behaviour^8^ of CrSi, while the fitting of Eq. (1) to the $$\chi (T)$$ indicates that both the itinerant and localized behaviours are present in the compound. The effective magnetic moment defined as $$\mu _{eff}=\hbox {p}_{{eff}}$$ $$\mu _B$$^29^, where $$p_{eff}=\sqrt{8C}$$, comes out to be 1.63 $$\mu _B$$ per Cr ion. It is reported in literature that the itinerant moment corresponding to the SDW type order in pure Cr metal is $$\sim$$ 0.43 $$\mu _B$$^30^ while in other Cr based alloy it varies between 0.8 to 1.4 $$\mu _B$$^31^. On the other hand free $$\hbox {Cr}^{3+}$$ ion has the localized moment of $$\sim$$ 3.87 $$\mu _B$$. The value of effective moment in CrSi is found to be slightly higher than the itinerant moment as observed in other Cr based alloys. This is consistent with our itinerant electron magnetism picture in CrSi indicated by the finite positive value of $$\chi _0$$. The negative value of $$\theta$$ implies the presence of *AFM * correlations in the compound. The presence of these *AFM* correlations probably smears out the inflection point corresponding to the building-up of the *FM* correlations in CrSi, and makes the inflection point difficult to observe.

**Figure 2:**
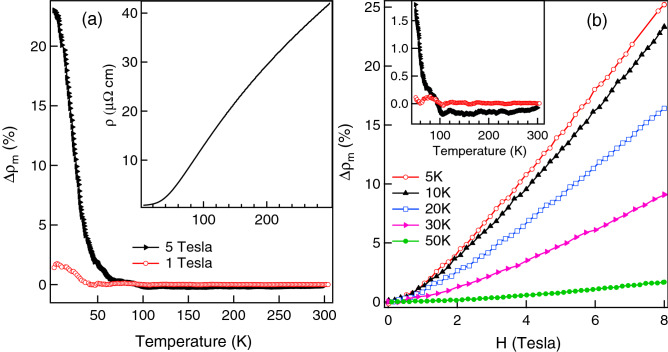
*MR* ($\Delta \rho _m$) as a function of temperature in 1 and 5 T magnetic fields are shown in (**a**). The zero field resistivity (*ρ*) as a function of temperature is shown in the inset of (**a**). *MR* ($\Delta \rho _m$) as a function of field (*H*) at different temperatures are shown in (**b**). Inset in (**b**) shows the zoomed *MR* data of (**a**) in the temperature range 50–305 K.

The *M* versus *H* behaviour of CrSi is shown in Fig. 1d. The curves do not saturate up to the highest applied field of 7 T at any temperature, indicating the absence of long range *FM* order in CrSi. Moreover, it is observed that *M* values measured at 30 K is less than those measured at 100 K and 50 K. It is also found that while the *M*(*H*) at 50 K is slightly non-linear, the *M*(*H*) at the other measured *T* (both higher and lower *T*) are very linear. We conjecture that there are competing *FM* and *AFM* correlations in CrSi. While the *FM* correlations develop in CrSi below about 165 K, the lowering of *M* with decreasing *T* in between 50 and 15 K may be due to *AFM* correlations.

The inset of Fig. 2a shows the resistivity ($\rho$) versus *T* behaviour of CrSi in zero magnetic field. CrSi shows a metallic character with a very small value of (residual) resistivity $\approx 0.8~\mu \Omega$ cm at 5 K. The main panel of Fig. 2a shows the *T* dependence of *MR* ($\Delta \rho _m\%$) in 1 and 5 T magnetic fields. *MR* is defined as $\Delta \rho _m(T,H)=~\frac{[\rho (T,H)-\rho (T,H=0)]}{(\rho (T,H=0))}\times 100 \%$, where $\rho (T,H)$ and $\rho (T,H=0)$ are the resistivities with and without magnetic field respectively. It is observed that CrSi has a positive *MR* below 80 K. The positive *MR* effect at low temperatures is enhanced from about $2\%$ in 1 T to about 25$\%$ in 5 T magnetic field (see Fig. 2a). It may be noted that the ($\chi -\chi _0$)$^{-1}$ versus *T* plot of Fig. 1c exhibits a change of slope initiating below $\sim$ 80 K, indicating the onset of new magnetic correlations as compared to the higher *T*. At higher *T*, the *MR* is very small and is in fact slightly negative as is observed more clearly for 5 T magnetic field (see inset to Fig. 2b). The positive *MR* in CrSi at low temperatures is also observed in the $\Delta \rho _m$ versus *H* curves shown in the main panel of Fig. 2b (at temperatures below 50 K), where a large positive *MR* of $\sim \,25\%$ is recorded at 5 K in 8 T magnetic field. The *MR* presented in Fig. 2a is somewhat higher than that of Fig. 2b. This is related to the fact that the measurements for the curves in Fig. 2a were performed using an AC excitation (14 Hz, in the PPMS) where the current through the sample is in a plane perpendicular to that of the applied magnetic field. The measurements for Fig. 2b, on the other hand, were performed using a DC, and the current and the magnetic field were in the same plane. It may be noted here that a pure *PM* metal without magnetic correlations generally exhibits a small positive *MR* in high *H* due to the orbital motion of the conduction electrons (Kohler rule)^32^. The small negative *MR* in CrSi at high *T* indicates that there are short range magnetic correlations within the *PM* phase of the present sample, and that the spin disorder scattering of conduction electrons is suppressed by the applied *H*. This further indicates that these short range magnetic correlations are of *FM* nature, which is commensurate with the inflection point observed in the *M*(*T*) curve and the deviation from linearity observed in the $(\chi -\chi _0)^{-1}$ versus *T* plot (see Fig. 1c). It may further be noted that a large positive *MR* has been reported in ordered *AFM* materials^33–37^, in the materials with *AFM* correlations within the *PM* phase^37^, and in *FM* compounds having hints of *AFM* fluctuations^38,39^. Moreover, the positive *MR* in the systems with *AFM* correlations has been explained theoretically^35,40–42^ as well. We believe that the large positive *MR* in CrSi is due to *AFM* correlations, and this is consistent with the large negative $\theta$ obtained from the analysis of the ($\chi -\chi _0)^{-1}$ versus *T* curve. Thus our *MR* results support our conjecture that CrSi is a compound with mixed magnetic interactions with signatures of competition between *AFM* and *FM* correlations, with the *FM* correlations being comparatively weaker. This weaker *FM* correlations and competition between *AFM* and *FM* interactions may be related to the presence of *DM* interaction in CrSi^8^.

**Figure 3:**
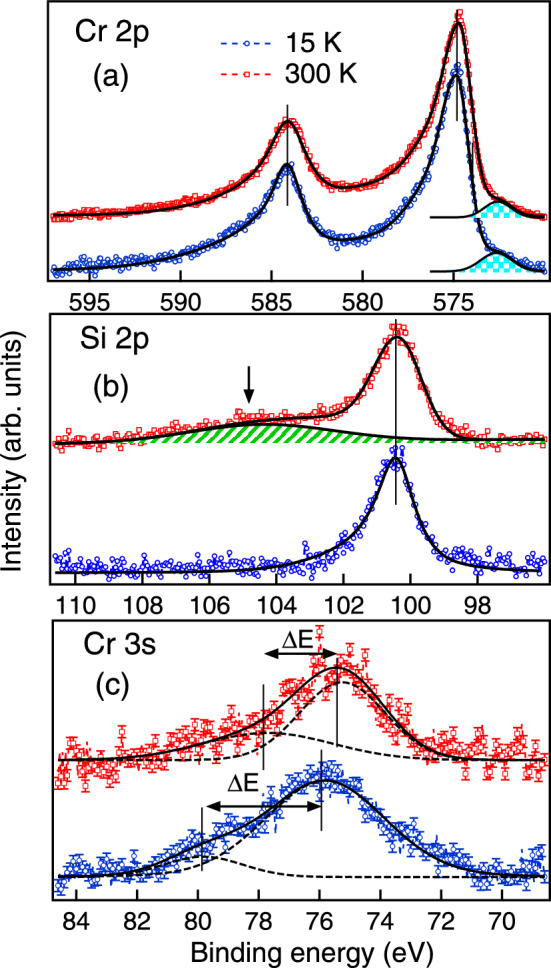
XPS core spectra at 300 K (RT) and 15 K (LT) showing (**a**) Cr 2*p*, (**b**) Si 2*p* and (**c**) Cr 3*s* levels. Solid lines in (a) and (b) are the fitting to the experimental data. Shaded region in (a) is the satellite feature in Cr *2p* core level. In (b) arrow shows the plasmon feature in Si *2p* core level. Solid and dashed lines in (**c**) show the total fitting to the Cr 3*s* spectra and the exchange split features, respectively.

### Electronic structure

To probe into the origin of magnetic interactions in CrSi, we have performed the core level studies at room temperature (RT = 300 K) and low temperature (LT = 15 K). Figure 3a–c show the Cr 2*p*, Si 2*p* and Cr 3*s* core levels. The inelastic background has been subtracted from raw data by the Tougaard procedure^43^. The spin-orbit splitting between Cr $2p_{3/2}$ and $2p_{1/2}$ in CrSi is 9.32 eV at both RT and LT, which is higher than that of elemental Cr $\sim$ 9.20 eV. A low energy satellite is observed in Cr 2*p* core level around $572.3\pm 0.1$ eV (shaded region in Fig. 3a). Similar low energy satellite has been observed in Mn 2*p* core level in $\hbox {MnSi}_{1.75}$^14^, Mn based Heusler alloys^44^ and $\hbox {La}_{0.7}\hbox {Sr}_{0.3}\hbox {MnO}_3$^45^, where this has been attributed to the delocalized type of screening of the $2\hbox {p}_{3/2}$ holes and the presence of delocalized states at $E_F$^45^. Such low energy satellites are also observed in rare-earth based intermetallics like $\hbox {CeAg}_2\hbox {Ge}_2$^26^ and PrGe^1^ where it arises due to strong hybridization of the localized 4*f* states with the conduction electrons. We find that the intensity of the satellite line increases with decreasing temperature which could be related to the change in hybridization due to the localization of Cr states at lower temperature. Similar changes in the satellite feature has also been observed in other transition metal silicides like $\hbox {MnSi}_{1.75}$ and CoSi^14^. The Si 2*p* core level in Fig. 3b shows a higher full width at half maximum at RT ($\approx ~1.65~\hbox {eV}$) as compared to that at the LT ($\approx ~1.5~\hbox {eV}$). In addition a broad plasmon feature (marked by arrow in Fig. 3b) is observed at $\sim \,105~\hbox {eV}$ in Si 2*p* peak at RT, that is found to be absent at LT. The origin of plasmon in Si at RT is because CrSi behaves like a heavily doped semiconductor where the metallic Cr states are hybridized with the semiconducting Si states. At RT the thermally excited conduction electrons in this heavily doped semiconductor gives rise to the plasmons while at LT the semiconductor like behaviour dominates which leads to the absence of the plasmon feature. Similar plasmon feature has been reported in the metal/insulator like interface in Si^46^. Such metallic states which lie in the band gap of semiconductors gives rise to the topological surfaces and is reported in CrSi^47^. The Cr 3*s* spectra are shown along with the error bars in Fig. 3c as the data is bit noisy due to the lower photoionization cross-section in the XPS measurements. From Cr 3*s* core level we have determined the exchange energy as well as the magnetic moment per Cr atom. The exchange splitting ($\Delta \hbox {E}$) was determined by fitting the peaks as shown in Fig. 3c. $\Delta \hbox {E}$ at LT $\approx ~4~\hbox {eV}$ is found to be higher than that at RT $\approx ~\hbox {2.4}~eV$, and this is in good agreement with the signatures of magnetic correlations found at low temperatures in the *M* and *MR* measurements. The magnetic moment can be determined from the intensity ratio (I) of the fitted peaks at LT and RT such that I= S/(S+1) where S is the spin and the magnetic moment is $M=~g\sqrt{S(S+1)}$. The value of *S* comes out to be $\sim$ 0.27 and $\sim$ 0.54 respectively at LT and RT. The magnetic moment determined per Cr atom at RT is $\approx$ 1.82 $\mu _B$ which decreases to $\approx$ 1.16 $\mu _B$ at LT. The value of the magnetic moment determined from the core level studies is in good agreement with that estimated from the magnetization measurement ($\approx \,1.63~\mu _B$).

**Figure 4:**
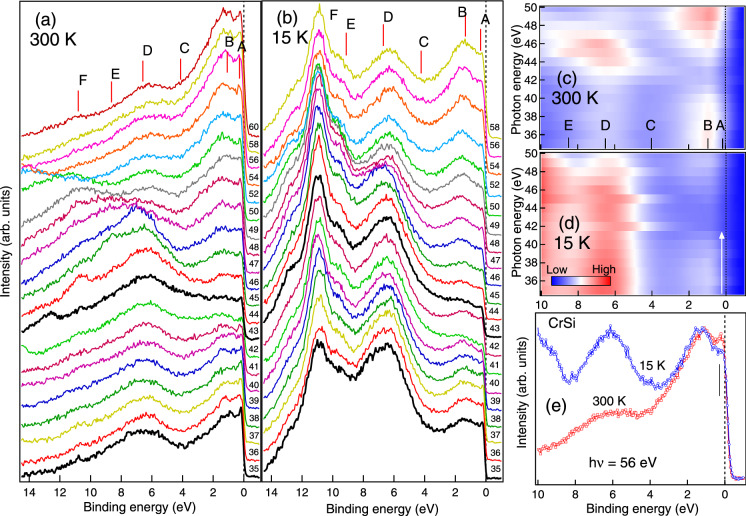
Resonant photoemission spectra (**a**) at 300 K and (**b**) at 15 K recorded across the Cr 3*p*–3*d* resonance. Valence band spectra are staggered for the clarity of presentation. Prominent features are marked in the spectra and the Fermi edge ($E_F$) is shown by dotted line at zero binding energy position. (**c**) and (**d**) show the contour plots of the RPES data where the colour scale represents the intensity. (**e**) shows the comparison of the 300 K and 15 K valence band spectra at 56 eV excitation energy.

To understand the nature of magnetic 3*d* electrons at RT and LT, we have performed RPES measurements across the 3*p* threshold of Cr in the photon energy range 35–60 eV which are shown in Fig. 4a, b respectively and the preliminary results reported in Ref.^48^. We find that the spectral shape of the RPES curves in the *PM* state at RT (Fig. 4a) is drastically different form that at LT (Fig. 4b). Six features are observed in the valence band spectra at 0.15, 0.95, 4, 6.5, 8.5 and 10.8 eV marked in the Fig. 4a, b as *A*, *B*, *C*, *D*, *E* and *F*, respectively. The features *A*, *B* and *C* in Fig. 4a, b have the dominant contribution from the Cr 3*d* states, while the feature *D*, *E* and *F* have mixed contribution from Cr 3*d*, Si 3*s* and Si 3*p* states^49^. On comparing Fig. 4a, b we find that the intensities of *D*, *E* and *F* enhance at LT while the intensity of *B* remains constant with varying *T*. The intensity of *A*, on the other hand, reduces at LT. The changes in the density of states are clearly visible in the contour plots shown in Fig. 4c, d. It is quite evident that the Cr 3*d* states (features *A* and *B*) become more localized at LT. Hence at RT, where the Cr 3*d* states are delocalized, we observe more dominating Cr 3*d* character while at LT a mixture of Cr and Si characters is observed. Increase in hybridization is therefore expected at LT since the reduced thermal energy causes finite overlap of the Cr 3*d* orbital with the Si 3*s* and 3*p* orbitals. As the 3*s*–3*p* bands in elemental Si are highly localized, the strong hybridization of the Cr and Si states at LT enhances the localization effect. We find that the localization effect causes a decrease in the partial density of states (PDOS) of the Cr 3*d* states (see arrow in Fig. 4d and tick in Fig. 4e) for feature *A* near Fermi edge ($E_F$ ). The observed phenomena related to the drastic modification in the density of states of CrSi is explained in detail later in the text.

**Figure 5:**
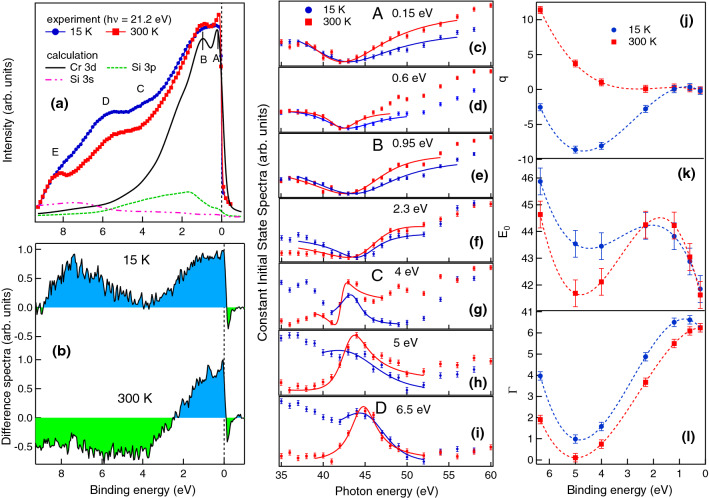
(**a**) High resolution valence band at RT and LT compared with the theoretical calculation as in Ref.^49^. (**b**) The difference spectra calculated by subtracting Off-resonance spectrum (35 eV) from On-resonance spectrum (43 eV). (**c**)–(**i**) show the CIS plots at different binding energies in the valence band mentioned in the plots. The solid lines in (**c**)–(**i**) show the fitting of the Fano line shape. The Fano parameters *q*, $E_0$ and $\Gamma$ are shown in (**j**), (**k**) and (**l**) respectively for RT and LT and the dash lines are guides to the eye.

High resolution valence band measurement using He I excitation energy ($h\nu = 21.2~\hbox {eV}$) also shows similar behaviour in the valence band at LT and RT as shown in Fig. 5a. We have compared our experimental data with the partial density of states (PDOS) of Cr 3*d*, Si 3*s* and Si 3*p* as reported in Ref.^49^. The PDOSs have been broadened by using the standard procedure reported elsewhere^1,26^. The features obtained in the valence band at RT shows a very good agreement with the reported theoretical calculation^49^. Comparing the calculated Cr 3*d* PDOS with the experimental valence band spectra, we find that the band width increases more at LT indicating an enhancement of the hybridization between Cr 3*d* and Si 3*s*–3*p* valence electrons at LT. Both the decrease in the intensity of the features near $E_F$ and the enhancement of the features away from $E_F$ at LT are mainly related to increased hybridization due to orbital overlap and change in the screening of the Cr 3*d* electrons by the conduction electrons.

Experimental Cr 3*d* PDOS can be determined from the difference of the On-resonance spectrum at 43 eV and the Off-resonance spectrum at 35 eV excitation energy as in Fig. 4a, b (shown by thick black lines). The difference spectra at the RT and LT phases are shown in Fig. 5b. The positive value in the difference spectrum indicates the dominating Cr 3*d* states while the negative value indicates the Si 3*s*–3*p* valence states. At RT we find that the Cr 3*d* states lie near the $E_F$ up to $\sim$ 2.5 eV while the Si 3*s*–3*p* character lies at higher binding energy above  $\sim$2.5 eV from the $E_F$. The higher density of delocalized Cr 3*d* states at the $E_F$ at RT is responsible for the origin of plasmonic excitation as discussed earlier and the signature of this phenomenon is observed in the Si 2*p* core level in Fig. 3b. However, at LT in Fig. 5b we find that there is a mixed Cr 3*d* and Si 3*s*–3*p* character all over the valence band. There are clearly two different characters of the hybridized Cr 3*d* states at LT, separated by a dip at $\approx$4 eV. The states which lie in the range up to $\approx$4 eV from $E_F$ are of more delocalized character while the states which lie above $\approx$4 eV have more localized character.

In order to clearly identify the nature of the Cr 3*d* states in the valence band, we have plotted the constant initial state (CIS) spectra in Fig. 5c–i for different binding energies in the range between $E_F$ to 6.5 eV. The CIS spectra have been determined from Fig. 4a, b by plotting the intensity at fixed binding energy positions in the valence band as a function of photon excitation energy. The CIS spectra for the features *A* and *B* in Fig. 5c, e, respectively show a resonance dip. While the features *C* and *D* in Fig. 5g, i, respectively show the resonant peaks. These electronic states have different mixing strengths between the localized and continuum at RT and LT. The CIS spectrum gives rise to a characteristic Fano line profile of the form $\sigma (h\nu )=\sigma _a \frac{(q+\epsilon )^2}{1+\epsilon ^2} + \sigma _b$ and $\epsilon =(h\nu -E_0)/\Gamma$^50^. Here, $E_0$ is the resonance energy and $\Gamma$ is the half-width of the line or the natural width given by the decay rate of the autoionization resonance. The Fano parameter *q* represents the discrete/continuum mixing strength, i.e. the coupling strength, and also determines the line shape. The parameters $\sigma _a$ and $\sigma _b$ represent the non-resonant background cross-sections for transitions to the continuum states that interact or do not interact, respectively, with the discrete autoionization states. In Fig. 5, the markers (filled circles and squares) and the solid line (red and blue) show the experimental data points and the fitted Fano line shapes, respectively. The parameters $\Gamma$, *q* and $E_0$ determined from the fitting are plotted in Fig. 5j–l, respectively. We find that the trend in $\Gamma$ and $E_0$ at RT and LT remains almost same for the features *A* and *B* near $E_F$ (Fig. 5k, l). However, at higher binding energy (for the features *C* and *D*) there is considerable variation in the value of $\Gamma$ and $E_0$ at LT and RT due to the change in hybridization strength. Interestingly in Fig. 5j we find that the value of *q* for the feature D is quite large and positive ($q =11.4$) at RT while the value of *q* is negative at LT ($q = -2.6$).

As stated above, the value of *q* gives the information about the coupling strength between the continuum and the discrete states present in the system. If the coupling strength between the continuum states and the discrete state is very weak, the value for *q* becomes large and a Lorentz line shape is expected. For a strong coupling strength, *q* is close to zero and one expects to see a resonance dip. For all other cases of the coupling strength, the variation in the cross section caused by a resonance can be described by a Fano-like line shape. If *q* is negative, the minimum in the absorption cross section occurs on the high-energy side of the resonance energy while for *q* positive, the minimum in the absorption cross section occurs on the low-energy side of the resonance energy. Nearly zero value of *q* for the features *A* and *B* indicates delocalized 3*d* electrons near $E_F$ that act as spin polarized conduction electrons. Thus the magnetism in CrSi is due to itinerant Cr 3*d* conduction electrons, and hence *AFM* correlations indicated in our *M* and *MR* is due to the SDW in CrSi. The large value of *q* for the *C* and *D* features indicates more localized valence electrons and the positive and negative *q* at LT and RT are related to the changes in hybridization between the Cr 3*d* and Si 3*s*–3*p* states due to the orbital overlap. Thus both the localized and itinerant characters are present in CrSi. We find that the exchange energy is less at RT where the density of localized states is less as compared to the itinerant states. The increase in the exchange energy at LT and the change in the density of delocalized states are also well corroborated in our core-level studies. The *d*-shells of CrSi exhibit quasi atomic character due to strong bonding of the *d* electrons with the Si lattice which is also reported in Ref.^28^. We find that the delocalized 3*d* electrons contributing to conduction are spin polarized by the interaction with the local 3*d* moment. The interaction between localized and delocalized electrons at LT leads to enhanced screening of the localized moment by the delocalized electrons which is clearly observed in the valence band. This seems to enhance the *AFM* correlations in CrSi at LT. Apart from the strong spin-orbit coupling observed in CrSi, and its crystal structure lacking inversion symmetry, the interaction between the moment carrying localized and itinerant Cr 3*d* electrons appear to be a crucial aspect in realizing the DM interaction in this system that leads to the mixed magnetic interactions with comparatively weaker *FM* correlations.

**Figure 6:**
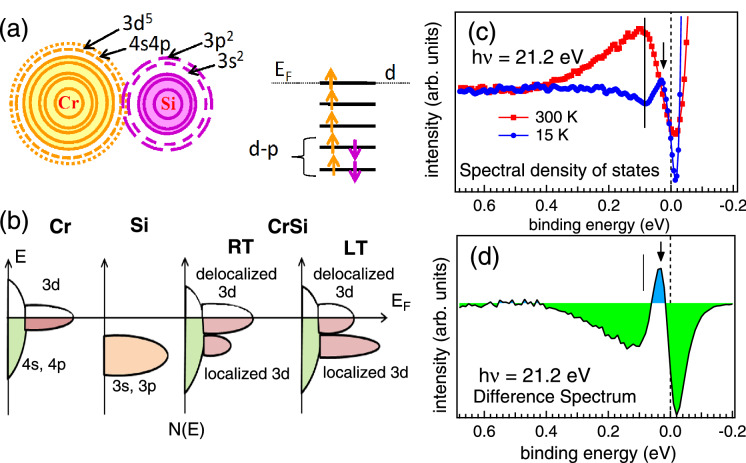
Schematic diagram showing the valence orbital overlap of Cr atom with its near neighbour Si atom and the energy level diagram for the $d-p$ hybridization in (**a**). In (**b**) the schematic diagram for the density of states N(E) as a function of E is shown for elemental Cr, elemental Si and CrSi compound at RT and LT phases. Spectral density of states at RT and LT derived from the high resolution valence band measured with He-I excitation are shown in (**c**). Difference spectrum between the RT and LT data obtained using He-I excitation is shown in (**d**). The pseudogap present in the system is shown by ticks in (**c**) and (**d**).

*DM* interaction is known to be associated with the topological phase transition^51,52^ where the topological edge states are likely to appear near $E_F$ within the bulk magnon gap^53^. The magnon gap is the signature of SDW which arises due to the electron-electron interaction^54^. In CrSi, the electron-electron interactions can be understood from the schematic diagrams shown in Fig. 6a, b. The Cr atom in CrSi has $3d^54s^1$ valence electrons and Si has $3s^23p^2$ valence electrons. Due to the near neighbour interaction in CrSi the valence orbitals of Cr overlap with the valence orbitals of Si as shown in Fig. 6a. At low temperatures the overlap of the orbitals is expected to increase, which leads to increase in hybridization and hence, the valence band broadening has been observed (in Fig. 5a). The elemental Cr atoms have initial mixing of the 3*d* and 4*s* orbitals through *s*–*d* hybridization as shown in the schematic diagram in Fig. 6b and the partially filled localized 3*d* states lie at and near the $E_F$. On the other hand, the 3*s*–3*p* orbitals of elemental Si are more localized in nature and lie well below $E_F$. In CrSi compound the strong $d-p$ hybridization leads to both localized and delocalized states (Fig. 6b). The localization occurs due to the spin pairing between the Cr 3*d* and the Si 3*p* electrons (Fig. 6a, see energy level diagram). Hence, the localization is mainly governed by the Si 3*p* states, while the delocalized states are mostly the Cr 3*d* states as shown in Fig. 6a (energy level diagram). We have observed a drastic variation in the density of states N(E) with temperature in CrSi in the experiments (Fig. 6b) where we find that there is a larger density of delocalized states at RT while at LT the density of delocalized states decreases with the enhancement of the localized states. Hence, the topological effects^47^ in this system are mainly governed by the Cr 3*d* states, as is explained below.

The topological properties depend on the change in the spectral density within a very narrow energy near $E_F$. Hence in Fig. 6c we have shown the spectral density of states (SDOS) in the valence band probed using very high energy resolution He-I radiation. The SDOS is extracted by dividing the experimental valence band data as obtained in Fig. 5a by the resolution broadened Fermi–Dirac distribution function at 300 K and 15 K. The SDOS shows a maximum in intensity at RT at $\sim \,80$ meV (shown by a vertical line in Fig. 6c) and a minimum in intensity at the same energy position at LT which is a clear signature of the pseudogap and is in consistent with the RPES results in Fig. 4d. It is reported that pseudogap arises in case of the SDW ordering in Cr metal^55^. We also find that a peak developed at 30 meV at LT (marked by arrow in Fig. 6c) is a signature of the topological states present at LT. The same topological peak is also present in the difference spectra in Fig. 6d (shaded blue peak) which is obtained by subtracting the valence band of 15 K from that of 300 K (Fig. 5a). The area of the difference spectra in Fig. 6d below the $E_F$ up to 0.4 eV at LT scales with the number of electrons getting excited at RT. Similarly, the area in the difference spectra (Fig. 6d) above the $E_F$ up to − 0.2 eV scales with the hole density of states getting occupied at RT. In normal metal these two areas are identical. We find in CrSi that the area above the $E_F$ is larger than the area below $E_F$. This observation clearly indicates the electron-hole asymmetry at LT along with the presence of pseudogap which indicates the evidence of topological phase transition in CrSi.

## Conclusion

Our magnetization and *MR* studies indicate that CrSi is a compound with competing *AFM* and *FM* correlations, where the *FM* correlations seem to be weaker. Our core-level studies indicates strong spin-orbit coupling in CrSi. We find that the delocalized Cr 3*d* conduction electrons are spin polarized by the interaction with the local Cr 3*d* moments. In the presence of large spin orbit coupling and the non-centrosymmetric crystal structure, the interaction between these localized and itinerant magnetic 3*d* electrons lead to *DM* interaction with competing *AFM* and *FM* correlations. CrSi exhibits a large positive *MR* which increases to $\sim \,25\%$ at 5 K in 5 T magnetic field. From Cr 3*s* core level studies we find that the exchange splitting at 15 K ($\approx ~4~\hbox {eV}$) is higher than that at 300 K ($\approx$ 2.4 eV), and that the magnetic moment per Cr atom is $\approx$ 1.82 $\mu _B$ at 300 K and $\approx ~1.16~\mu _B$ at 15 K which is in good agreement with the magnetization measurements ($\approx$1.63 $\mu _B$). Both the magnetization and the electronic structure indicate that the magnetic moment in CrSi is partially itinerant and partially localized. Our RPES measurements indicate that while the Cr 3*d* states of CrSi are quite delocalized at 300 K, they become more localized at 15 K where strong hybridization between Cr and Si states enhances the localization. Our high resolution valence band measurements show a valence band broadening at LT, indicating an enhancement of the hybridization between Cr 3*d* and Si 3*s*–3*p* valence electrons at LT. Analysis of CIS spectra indicates itinerant magnetism in CrSi due to Cr 3*d* conduction electrons, and hence the *AFM* correlations in CrSi is SDW type. Spectral density of states derived from high resolution valence band measurements provides evidence of the electronic topological phase transition in CrSi. Large density of itinerant electrons along with *AFM* correlations, large *MR*, etc. are currently desired for spintronics applications. Additionally, appreciable variation in the measured density of states of our CrSi sample at the Fermi edge with varying temperature shows the potential of CrSi for thermoelectric applications.

## Methods

### Sample preparation and characterization

Polycrystalline ingot of CrSi was prepared by arc melting 99.999$\%$ pure constituent elements in Ar atmosphere. A small pellet was cut from the ingot for the photoemission measurements. X-ray diffraction (XRD) measurements were performed on the same bulk pellet. The XRD pattern was recorded in a commercial powder X-ray Diffractometer (Bruker, model D8 Advance) using the characteristic Cu $K_\alpha$ radiation. Le Bail decomposition of the XRD pattern was performed using the JANA2000 package^56^.

### Magnetization and magnetoresistance

The temperature (*T*) and field (*H*) dependence of magnetization (*M*) were measured using a superconducting quantum interference device based vibrating sample magnetometer (MPMS-3 SQUID VSM, QuantumDesign). The magnetoresistance measurements as a function of temperature was performed using the AC transport option of a physical properties measurement system (PPMS, Quantum Design), and the magnetoresistance measurements as a function of field was performed using the DC transport method in a superconducting magnet-cryostat system from Oxford Instruments.

### Resonant photoemission

The resonant photoemission (RPES) measurements were performed at the Angle Resolved Photoelectron Spectroscopy beamline at Indus-1 Synchrotron^57^. The base vacuum during the measurement was $\sim \,3~ \times 10^{-10}$ mbar. The samples were cleaned by mechanical scrapping using diamond file. The absence of Carbon 1*s* peak at 284 eV and Oxygen 1*s* peak at 531 eV was ensured before the measurements. The valence band (VB) photoemission spectra were recorded using Phoibos 150 electron energy analyser and by varying photon excitation energies from 35 to 60 eV across the Cr 3*p*–3*d* transition and with the typical energy resolution of 135 meV.

### High resolution valence band and X-ray photoemission

High resolution VB measurements with 10 meV energy resolution at 15 K and 100 meV energy resolution at 300 K were performed using the monochromatic He-1 line from SPECS UVS 300 source and SPECS Phoibos 150 electron energy analyser at the experimental station of angle resolved photoelectron spectroscopy beamline at Indus-2 Synchrotron. High resolution VB spectra were recorded with a SPECS Phoibos 150 electron energy analyser. Core levels were studied using X-ray photoemission spectroscopy (XPS) with Mg $K_\alpha$ source from SPECS (XR 50). The base vacuum during the measurement was $7\times 10^{-11}$ mbar.
